# A Topology Reorganization Scheme for Reliable Communication in Underwater Wireless Sensor Networks Affected by Shadow Zones

**DOI:** 10.3390/s91108684

**Published:** 2009-10-29

**Authors:** Mari Carmen Domingo

**Affiliations:** Department of Telematics Engineering, Barcelona Tech University, Esteve Terradas, 7 - 08860 Castelldefels (Barcelona), Spain; E-Mail: cdomingo@entel.upc.edu; Tel.: +34-93-413-70-51; Fax: +34-93-413-70-07

**Keywords:** underwater sensor networks, shadow zones, transmission loss, optimal location

## Abstract

Effective solutions should be devised to handle the effects of shadow zones in Underwater Wireless Sensor Networks (UWSNs). An adaptive topology reorganization scheme that maintains connectivity in multi-hop UWSNs affected by shadow zones has been developed in the context of two Spanish-funded research projects. A mathematical model has been proposed to find the optimal location for sensors with two objectives: the minimization of the transmission loss and the maintenance of network connectivity. The theoretical analysis and the numerical evaluations reveal that our scheme reduces the transmission loss under all propagation phenomena scenarios for all water depths in UWSNs and improves the signal-to-noise ratio.

## 1. Introduction

**Figure 1.**
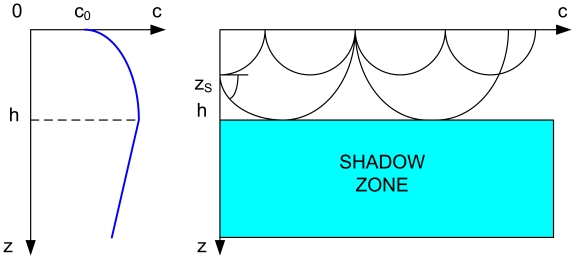
Shadow zone formation beneath the mixed layer when sound velocity monotically decreases with depth.

Underwater Wireless Communication Networks (UWCNs) are formed by sensors and Autonomous Underwater Vehicles (AUVs) interacting together to perform specific underwater applications such as collaborative monitoring or surveillance [1]. Communication quality in Underwater Wireless Sensor Networks (UWSNs) is very challenging due to the harsh characteristics of the underwater channel, such as high and variable propagation delays, limited bandwidth, high bit error rates, multipath phenomena and multipath fading [1]. In the extreme case, the spatially-variant underwater channel can cause the formation of shadow zones, which are time-variant areas where there is little signal propagation energy due to the refraction of signals by the sound speed fluctuation [2]. When the sound speed has a negative gradient just beneath the surface [3], a shadow zone is formed because the acoustic rays are refracted downward. Refraction produces shadow zones that sound waves do not penetrate because of their curvature. The sea bottom can produce a shadow zone as well, when the rays are refracted upward. Shadow zones [3] can also appear beneath the mixed layer for a source located near the ocean surface because the acoustic energy is trapped in the surface duct (see Figure 1). The shadow zone is usually bounded by the lower boundary of the surface duct and the limiting ray. Shadow zones can also appear between convergence zones. If the source is located at the same depth of the underwater sound channel axis, the shadow zone will disappear [4]. We distinguish between shallow (depth up to 100 m) and deep water. In shallow water (order of 100 meters depth) and at ranges of 3 kms shadow zones appear [2]. In deep water (order of 1,000s of meters depth) and at ranges of 10s of kms shadow zones are formed [2].

Shadow zones cause high bit error rates, losses of connectivity and dramatically impact communications performance. Some experiments show that for high frequencies signal levels are typically at least 40 dB less than those at the edges of the shadow zone [5]. For low frequencies the signal loss in the shadow zone is less severe; however, variations in received signal-to-noise ratios (SNRs) by as much as 10 dB have been observed on time scales of several hours [6]. Therefore, many authors recommend devising solutions to handle their effects [7,8].

For these reasons, in this paper we focus on communication reliability in the presence of shadow zones. We propose a distributed adaptive topology reorganization scheme that alleviates the effects of energy limitations and is able to maintain connectivity between sensor nodes in multi-hop three-dimensional UWSNs in the presence of shadow zones. Besides, it is able to estimate when the shadow zones have disappeared using double sensor units to restablish communication very quickly through the original acoustic wireless links. We study the effects of the proposed scheme in shallow (depth up to 100 m) and deep water. A two-path Rayleigh fading channel model and different propagation phenomena are considered: shallow water, deep water with convergence zones, deep water with deep sound channel and shallow or deep water with shadow zones.

According to the number of links in the three-dimensional UWSN affected by the shadow zone, three different cases have been introduced. In this paper we have extended our work in [9], where only one case was discussed. A mathematical model has been developed for each case to find the optimal placement for the sensor nodes whose ongoing communications are being disturbed. The two major objectives of the proposed mathematical model are: the minimization of the transmission loss and the maintenance of network connectivity. To the best of our knowledge this is the first paper that finds the optimal location for underwater sensor nodes affected by a shadow zone under different propagation phenomena.

The theoretical analysis and the numerical evaluations reveal that the average transmission loss values are reduced significantly under all propagation phenomena for all water depths in UWSNs when the optimal locations are computed and the communication between sensor nodes is again restablished outside the shadow zone. The average SNR values have also improved significantly and are maintained for all frequencies.

The remainder of the paper is organized as follows: In Section 2, we discuss the related work. In Section 3, we analyze our system model. In Section 4, we state the location optimization problems and propose nonlinear programming (NLP) formulations. In Section 5 we present our numerical results. Finally, we draw the conclusions in Section 6.

## 2. Related Work

Shadow zones represent a serious obstacle for good communication in UWSNs, because they cause network partition. Therefore, the proposal of solutions to handle their effects has been encouraged [7,8]. A distributed reactive shadow zone and delay aware (SZODAR) routing protocol for three-dimensional UWSNs was introduced in [10]. In SZODAR, the depth of the acoustic transceivers of sensor nodes is changed to avoid shadow zones, while the sensing modules are kept unchanged. During the connection establishment process, each node should send HELLO messages until it receives a routeReply or until the maximum number of allowed HELLO messages maxTrial is reached. If the number of HELLO messages is over maxTrial, the node is considered to be out of range or located in a shadow zone. In this case the node should increase its transmission power to the next power level stepwise up to the maximum and try to establish connection again at each step. Since UWSNs have very limited resources, the proposed connection establishment process consumes excessive power and can lead easily to energy depletion. In addition, it is not possible to determine if the node unable to establish connection using maximum power level is located in a shadow zone or is simply a node out of the range of the other nodes. Our proposed scheme has been designed to detect shadow zones based on the estimation of the transmission loss values, which is a not such an excessive energy consuming operation.

In addition, in [10] the acoustic transceiver of the node out of range or located in a shadow zone is moved upwards or downwards according to a random probability, whereas the node periodically tries to establish connection with its neighbours. If the boundary for the operational depth is reached and the connection establishment process has failed, the node moves in the opposite direction. With this arbitrary movement criterion this shadow zone awareness routing protocol consumes excessive power and incurs high delays and routing overhead. Moreover, the effects of different underwater propagation phenomena on the performance of the proposed protocol have not been analyzed.

In our proposed scheme sensor nodes are double units operating as a single sensor; they are decoupled into two sensor nodes in the presence of a shadow zone. One sensor node remains in the same position and estimates, as opposed to SZODAR [10], when the shadow zone has disappeared. An optimal placement imposing a restriction in energy consumption is computed for the other sensor node; this node is moved directly to this optimal location away from the shadow zone. In this way, connectivity between both sensors inside the shadow zone is established by a wire, connectivity between neighbour nodes is maintained and latency as well as power consumption are reduced. The effects of the different propagation phenomena on the performace of the proposed scheme have been analyzed. Furthermore, connection can again be reestablished very quickly using the original wireless acoustic links if the shadow zones disappear.

Several papers have proposed options related to location optimization of sensor nodes underwater. In [11], a placement scheme has been introduced to find the optimal placement of data collectors in UWSNs. In [12], a distributed node deployment technique for UWSNs to improve coverage and provide connectivity with the surface stations has been proposed and compared in terms of coverage and connectivity with the optimal centralized solutions from the literature. In [13], deployment strategies for two-dimensional and three-dimensional communication architectures for UWSNs have been developed to determine the minimum number of sensors to be deployed to achieve optimal sensing and communication coverage according to the application requirements. However, these contributions don't analyze which is the optimal location for underwater sensor nodes affected by a shadow zone under different propagation phenomena.

## 3. System Model Analysis

**Figure 2.**
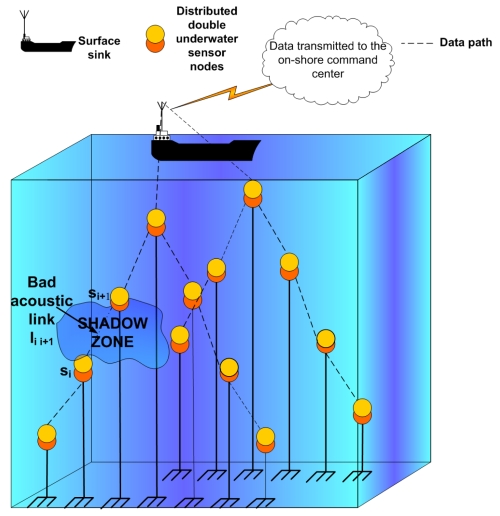
Architecture for a 3D underwater sensor network.

We consider a three-dimensional underwater sensor network for environmental monitoring (see Figure 2). In this architecture, a certain number of underwater sensors are deployed to cover a large area of a lake or ocean. Metal pieces at the seabed and thin ropes are used to attach the sensor nodes and distribute them at different depth levels [1]; they are deployed at different water depths to observe water quality parameters since water characteristics vary mostly in vertical direction. An electronically controlled engine at the sensors is used to change their depths [1]. This deployment strategy requires building a tree-like hierarchical multi-hop routing topology. The sensed data is sent to the surface station, which floats on the water surface and uses long distance radio communication to send this data to an onshore station. The onshore station is located at the monitoring center and performs further analysis of the data.

## 4. Problem Formulations and Optimization Models

We consider the example of the tree-like topology shown in Figure 2, where sensor nodes sense data and sent it upwards to the surface station using multi-hop routing. We assume that the distributed sensor nodes are double units. The cost of implementing double sensors will be limited because software modems will be coupled with generic microphones and speakers that are built-in to create off-the-shelf sensor modules [14-16]. In this way, the need for specialized communication hardware will be eliminated and the system cost will be reduced. The cost per node ($) for hardware modems that use especialized hardware is around 10^4^ $, whereas the cost of software modems that use generic hardware (off-the-shelf configuration) is around 10^2^ $; this cost decrease facilitates the deployment of sensor nodes to form underwater acoustic sensor networks. The double sensor nodes operate as a single sensor when the underwater communication is reliable. This fact changes in the presence of shadow zones.

Each intermediate node *s_i_* should be able to detect if the quality of the uplink *l_i i_*_+1_ towards one-hop neighbor *s_i_*_+1_ is affected by the presence of a shadow zone computing the transmission loss *TL_i i_*_+1_ [3]. If *TL_i i_*_+1_(*t* + Δ*t*) = *TL_i i_*_+1_(*t*) + *TL_shadow zone_* and this value persists during a period longer than *T* to eliminate insignificant variations, the sensor node assumes that the uplink is located in a shadow zone because *TL_i i_*_+1_(*t* + Δ*t*) has been increased by the transmission loss value *TL_shadow zone_* inside the shadow zone.

We distinguish between three different cases according to the number of links in the underwater sensor network affected by the shadow zone:

- Case 1: Only the uplink *l_i i_*_+1_ between *s_i_* and *s_i_*_+1_ is located in the shadow zone.
- Case 2: The uplink *l_i i_*_+1_ between *s_i_* and *s_i_*_+1_ and the uplinks *l*_(_*_i-_*_1)_*_k i_* between *s*_(_*_i-_*_1)_*_k_*, ∀*k* ∈ *K* and *s_i_* are located in the shadow zone. *K* represents the set of neighbours connected to *s_i_* through uplinks using hierarchical routing.
- Case 3: The uplink *l_i i_*_+1_ between *s_i_* and *s_i_*_+1_ and the uplinks *l*_(__*i*__-1)_*_k i_* between *s*_(_*_i-_*_1)_*_k_*, ∀*k* ∈ *K* and *s_i_, l*_(_*_i-_*_2)_*_k i-_*_1_ between *s*_(_*_i-_*_2)_*_k_, ∀k* ∈ *K* and a particular *s*_(_*_i-_*_1)_*_k_*, …, *l*_(_*_i-x_*_)_*_k i-x_*_+1_ between *s*_(_*_i-_*_x)_*_k_*, ∀*k* ∈ *K, ∀x* ∈ *N* and a particular *s*_(_*_i-x_*_+1)_*_k_* are located in the shadow zone.

Each case will be solved differently. In all cases we assume that the sensor node *s_i_* knows the location and dimensions of the shadow zone. The sound speed profile is affected by the time of the day, weather, depth, variations in the water temperature and salinity. With the aid of a forecast model [8] and the estimation of the sound speed profile, the different underwater propagation phenomena can be predicted, including the presence of shadow zones. The location of shadow zones can be performed studying the trajectory of the acoustic rays under the different underwater propagation phenomena [3]. Autonomous Underwater Vehicles (AUVs) can analyze and inform underwater sensors periodically about the existence and location of shadow zones.

We assume that sensor nodes are located in a grid (see Figure 6). The axes of the coordinate system are set at the water surface. Sensors know their fixed two coordinates (*x,y*). They are also aware of their depth (*z* coordinate), which varies with time depending on the networks tasks. The *z* coordinate is zero at surface and increases with depth. Sensor nodes also know the location of their neighbours because they exchange their depth information.

### Case 1: Only the uplink *l_i i_*_+1_ between s_i_ and *s_i_*_+1_ is located in the shadow zone

**Figure 3.**
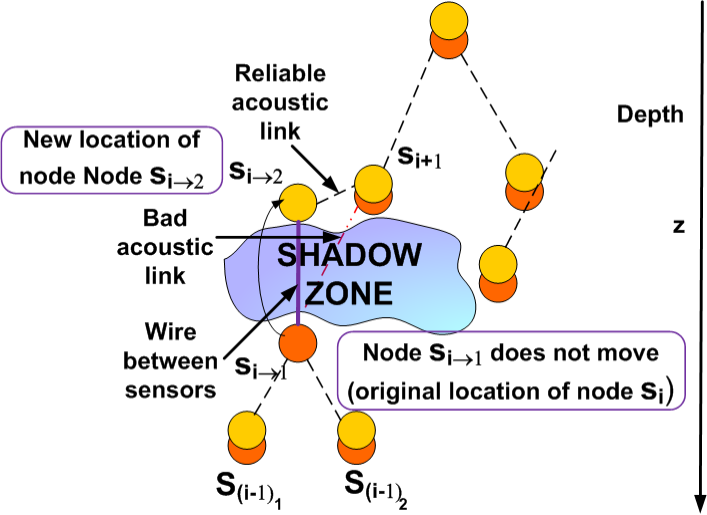
Scenario in the 3D UWSN with a shadow zone (Case 1).

In this case under the presence of a shadow zone the sensor node *s_i_* is uncoupled into two sensor nodes *s_i_*_→1_ and *s_i_*_→2_ as shown in Figure 3. These sensor nodes will be connected to each other inside the shadow zone by a wire to maintain robust communication. The node *s_i_*_→1_ remains in the same location as the node *s_i_* was and continues sensing data at the required depth level. We should now determine the optimal location for the node *s_i_*_→2_ We formulate this problem as a Nonlinear Program (NLP).

We introduce the following notation:

- *u_i_*_+1_ = (*x_i_*_+1_, *y_i_*_+1_, *z_i_*_+1_) is the location of node *s_i_*_+1_.
- *u_i_*_→2_ = (*x_i_, y_i_, z_i_*_→2_) is the new location of node *s_i_*_→2_.
- *H* is the sea depth.
- *R_MAX_* is the maximum transmission range.
- *TL_Th_* is the detection threshold.
- *r_i_*_→2 *i*__+1_ refers to the transmission range of the acoustic link between nodes *s_i_*_→2_ and *s_i_*_+1_ expressed in meters.
- α represents the absorption coefficient and has the units dB/Km.
- *χ* is a variable used to compute the transmission loss with the value *χ* = 10 for shallow water (cylindrical spreading) and *χ* = 20 for deep water (spherical spreading) [3].
- *A* is called the transmission loss anomaly and summarizes the combined effects of several complex factors such as multiple path propagation due to the variations of the speed with temperature, depth and salinity, refraction effects, diffraction and scattering of sound by particles, bubbles and plankton within the water column. Detailed formulas of the multipath propagation loss according to the different propagation phenomena (surface reflections, bottom reflections, convergence zones, deep sound channel, etc.) can be found in [3]. It is given in dB.
- *w_sz_*_−_*_start_* = (*x_i_, y_i_, z_sz_*_−_*_start_*) and *w_sz_*_−_*_end_* = (*x_i_, y_i_, z_sz_*_−_*_end_*) are the coordinates of the points delimiting the shadow zone at (*x_i_, y_i_*) with *z_sz_*_−_*_start_* < *z_sz_*_−_*_end_* and height *z_sz_*_−_*_end_* − *z_sz_*_−_*_start_*.
- *E_Bw_* is the energy consumed moving the sensor *s_i_*_→2_.
- *E_Th_* is the energy consumption threshold.

The problem can be formulated as follows:

#### P1: Location Optimization Problem

*Given*:*x_i_, x_i_*_+1_, *y_i_, y_i_*_+1_, *z_i_*_+1_, *H, R_MAX_,TL_TH_, α, χ*,*A, z_sz_*_–_*_start_, z_sz_*_–_*_end_, E_bw_, E_th_**Find:**z_i_*_→2_ ∈ [0, *H*], *r_i_*_→2 *i*__+1_ ∈ *R*^+^*Min:**TL_i_*_→2 *i*__+1_ = *χ* log_*i*__→2 *i*__+1_+*α.r_i_*_→2 *i*__+1_ 10^−3^ + *A**Subject to:*

(1) $$r_{i\to 2i+1}=\left\|u_{i+1}-u_{i\to 2}\right\|$$

(2) $$r_{i\to 2\;i+1}<R_{\text{MAX}}$$

(3) $$TL_{i\to 2\;i+1}<TL_{Th}$$

(4) $$z_{i\to 2}<z_{sz–\text{start}}$$

(5) $$E_{Bw}<E_{Th}$$

The objective function of problem **P1** aims at finding the best location for *s_i_*_→2_ that minimizes the transmission loss and preserves the connectivity with node *s_i_*_+1_ preventing from network partition.

The absorption coefficient α is computed as derived in [17,18] as a function of the frequency *f*, the salinity *S*, the temperature *T*, the water ph *pH* and the depth *z*. We consider a two-path Rayleigh fading channel model, where each path is Rayleigh distributed. More specifically, the envelope of the signal from each path in the two-path model is modeled as an independent Rayleigh distributed random variable, *α_i_, i* ∈{1,2} [19]. Consequently, for each path, the received energy per bit per noise power spectral density is given by, *ψ* = *α*^2^.(*E_b_/N_0_*) which has a distribution, *f*_Γ_(*ψ*)=(1/*ψ_0_*). *exp*(*-ψ/ψ_0_*) where *ψ_0_* = E[*α*^2^].(*E_b_/N_0_*) and *E_b_/N_0_* can be found from the SNR of the channel. Since the 2-path Rayleigh model does not have a closed-form expression for SNR distribution, it is found through simulations [19]. The value of *α* is used to find the transmission loss and the SNR of the channel.

Constraint (2) imposes that the transmission range should be lower than a threshold to ensure connectivity. Constraint (3) expresses that the transmission loss should be lower than a transmission loss threshold, that is, the maximum propagation loss for properly receiving the transmitted signal.

The SNR of an emitted underwater signal at the receiver can be expressed in dB by the passive sonar equation [ 3]:

(6) $$\psi_{0,dB}=SL-TL-NL+DI\geq \psi_{Th}$$

where *ψ_Th_* has been defined as the detection threshold, *SL* is the source level, *TL* is the transmission loss, *NL* is the noise level and *DI* is the directivity index.

The signal level *SL* is related to the intensity *I_t_* and hence to the transmission power of the transceiver for shallow water as follows:

(7) $$I_{t}=\frac{P_{t}}{2\pi \;1m\;z},SL=10\log\left(\frac{I_{t}}{0.67\times 10^{-18}}\right)$$

where *P_t_* is the transmission power in watts and *z* is the depth in meters.

In deep water the equation (7) becomes:

(8) $$I_{t}=\frac{P_{t}}{4\pi \;1m},SL=10\log\left(\frac{I_{t}}{0.67\times 10^{-18}}\right)$$

In our analysis we assume a fixed detection threshold for the received SNR. Based on this assumption we define the transmission loss threshold. It is given by [20]:

(9) $$TL_{Th}=\frac{SL}{NL\cdot \psi_{Th}}$$

where *SL* is fixed and *NL* is also fixed for a particular operating frequency.

We consider five different channel models:

- Shallow water.
- Deep water with convergence zones.
- Deep water with deep sound channel.
- Shallow water with shadow zones.
- Deep water with shadow zones.

Details of these models can be found in [3].

Constraint (4) imposes that the sensor node *s_i_*_→2_ should be located outside the shadow zone.

Constraint (5) states that the energy consumed by the electronically controlled engine to move the sensor to the optimal position should be lower than a threshold. It is defined as [21]:

(10) $$E_{Bw}=P_{Bw}.T_{Bw}+\lambda .v_{s}.T_{Bw}$$

where *P_Bw_* is the consumed power to move the sensor upwards or downwards and depends on the power required to drive the electronics, *T_Bw_* is the time required for the movement, *v_s_* is the moving speed and *λ* is a constant depending on factors such as the drag force, sensor size and shape, friction of the motor, etc. With the proposed mechanism some extra energy will be consumed to locate the shadow zone and to relocate the sensor to the required position, but communication in the shadow zone will be possible.

### Case 2: The uplink l*_i i_*_+1_ between *s_i_* and *s_i_*_+1_ and the uplinks *l*_(_*_i-_*_1)_*_k i_* between *s*_(_*_i-1_*_)_*_k_*, ∀*k ∈ K* and *s_i_* are located in the shadow zone. *K* represents the set of neighbours connected to *s_i_* through uplinks using hierarchical routing

**Figure 4.**
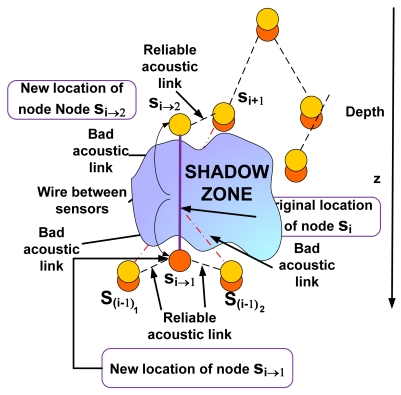
Scenario in the 3D UWSN with a shadow zone (Case 2).

In this case under the presence of a shadow zone the sensor node *s_i_* is uncoupled into two sensor nodes *s_i_*_→1_ and *s_i_*_→2_, as shown in Figure 4. These sensor nodes will be connected to each other inside the shadow zone by a wire to maintain robust communication. Now we should determine the optimal location for the nodes *s_i_*_→1_ and *s_i_*_→2_ using NLP. Since the topology is tree-like, we assume several nodes *s*_(_*_i-_*_1)_*_k_*, ∀*k* ∈ *K* forward their data messages towards node *s_i_*. Our objective consists of minimizing the transmission loss while preventing from network partition. For this purpose the connectivity between nodes *s_i_*_→1_ and *s*_(_*_i-_*_1)_*_k_*, ∀*k* ∈ *K* on the one hand, and the connectivity between nodes *s_i_*_→2_ and *s_i_*_+1_ on the other hand should be maintained. The optimal location for the node *s_i_*_→2_ is obtained solving the **P1** optimization problem already introduced in Case 1. We should now obtain the optimal placement for the node *s_i_*_→1_.

We define:

- *u_i_*_→1_ = (*x_i_, y_i_, z_i_*_→1_) is the new location of node *s_i_*_→1_.
- *u*_(_*_i-_*_1)_*_k_* = (*x*_(_*_i-_*_1)_*_k_, y*_(_*_i-_*_1)_*_k_, z*_(_*_i-_*_1)_*_k_*), ∀*k* ∈ *K* is the location of node *s*_(_*_i-_*_1)_*_k_*.

*Given:**x_i_, x*_(__*i*__−1)_*_k_, y_i_, y*_(__*i*__−1)_*_k_, z*_(__*i*__−1)_*_k_, H, R_MAX_,TL_Th_*,*α, χ, A, z_sz_*_–_*_start_, z_sz_*_–_*_end_, E_bw_, E_Th_**Find:**z_i_*_→1_ ∈ [0, *H*], *r*_(__*i*__−1)_*_k i_*_→1_ ∈ *R*^+^*Min* max*_k_ TL*_(__*i*__−1)_*_k i_*_→1_*Subject to:*

(11) $$r_{{\left(i-1\right)}_{k}i\to 1}=\left\|u_{i\to 1}-u_{{\left(i-1\right)}_{k}}\right\|,\forall k\in K$$

(12) $$r_{{\left(i-1\right)}_{k}i\to 1}<R_{\text{MAX}},\forall k\in K$$

(13) $$TL_{{\left(i-1\right)}_{k}i\to 1}<TL_{Th},\forall k\in K$$

(14) $$z_{i\to 1}>z_{sz–\text{end}}$$

(15) $$E_{Bw}<E_{Th}$$

#### P2: Location Optimization Problem

The objective function of problem **P2** aims at finding the best location for *s_i_*_→1_ that minimizes the transmission loss of the link *l*_(_*_i-_*_1)_*_k i_* with the highest transmission loss value and preserves the connectivity with nodes *s*_(_*_i-_*_1)_*_k_* preventing from network partition.

Constraint (12) imposes that the transmission range should be lower than a threshold to ensure connectivity. Constraint (13) expresses that the transmission loss should be lower than a transmission loss threshold. Constraint (14) imposes that the sensor node *s_i_*_→1_ should be located outside the shadow zone. Constraint (15) states that the energy consumed by the electronically controlled engine to move the sensor to the optimal position should be lower than a threshold.

### Case 3: The uplink *l_i i_*_+1_ between *s_i_* and *s_i_*_+1_ and the uplinks *l*_(i-1)_*_k i_* between *s*_(_*_i-1_*_)_*_k_*, ∀*k* ∈ *K* and *s_i_, l*_(__*i*__-2)_*_k i_*_-1_ between *s*_(_*_i-2_*_)_*_k_*, ∀*k* ∈ *K* and a particular *s*_(_*_i-_*_1)_*_k_*, …, *l*_(__*i*__-_*_x_*_)_*_k i-x_*_+1_ between *s*_(_*_i-x_*_)_*_k_*, ∀*k* ∈ *K, ∀x* ∈ *N* and a particular *s*_(_*_i-x_*_+1)_*_k_* are located in the shadow zone

**Figure 5.**
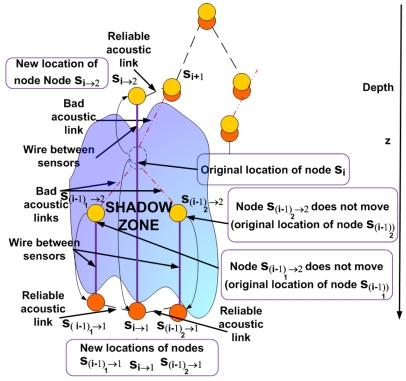
Scenario in the 3D UWSN with a shadow zone (Case 3).

In this case the sensor node located at the lowest depth in the shadow zone *s_i_* is uncoupled into two sensor nodes *s_i_*_→1_ and *s_i_*_→2_ as shown in Figure 5.

The optimal placement for node *s_i_*_→2_ is obtained solving the **P1** optimization problem already introduced in Case 1. The node *s_i_*_→1_ should move to (*x_i_, y_i_, z_sz-end_* + *ε*), where ε ∈ *R*^+^ is a very small value, that is, *s_i_*_→1_ moves vertically downwards outside the shadow zone. In this case, the connectivity with the nodes *s*_(_*_i-_*_1)_*_k_*, ∀*k* ∈ *K* through the uplinks can only be maintained if they also move outside the shadow zone. Therefore, the sensor nodes *s*_(_*_i-_*_1)_*_k_*, ∀*k* ∈ *K* are uncoupled into two sensor nodes *s*_(_*_i-_*_1)_*_k_*_→1_ and *s*_(__*i*__-1)_*_k_*_→2_. The node *s*_(_*_i-_*_1)_*_k_*_→2_ remains in the same location as node *s*_(_*_i-_*_1)_*_k_*, ∀*k* ∈ *K* was and continues sensing data at the required depth level. We should obtain the optimal placement for the node *s*_(_*_i-_*_1)_*_k_*_→1_. The data sensed by *s*_(_*_i-_*_1)_*_k_*_→2_ is sent through a wire between *s*_(_*_i-_*_1)_*_k_*_→2_ and *s*_(_*_i-_*_1)_*_k_*_→1_ to maintain communication inside the shadow zone. Generally speaking, the same process should be repeated for all the nodes down in the hierarchy *s*_(_*_i-x_*_)_*_k_*, ∀*k* ∈ *K*, ∀*x* ∈ *N* located in the shadow zone. They are uncoupled into two sensor nodes *s*_(__*i*__-_*_x_*_)_*_k_*_→1_ and *s*_(_*_i-x_*_)_*_k_*_→2_. The node *s*_(_*_i-x_*_)_*_k_*_→2_ remains in the same location as node *s*_(__*i*__-_*_x_*_)_*_k_*, ∀*k* ∈ *K*, ∀*x* ∈ *N* was and continues sensing data at the required depth level. We should obtain the optimal placement for the node *s*_(_*_i-x_*_)_*_k_*_→1_. The data sensed by *s*_(_*_i-x_*_)_*_k_*_→2_ is sent through a wire between *s*_(__*i*__-_*_x_*_)_*_k_*_→2_ and *s*_(*i-x*)*k*→1_ to maintain communication inside the shadow zone.

If the node *s*_(_*_i-x_*_+1)_*_k_*_→1_ has already moved to a new location outside of the shadow zone and node *s*_(_*_i-x_*_)_*_k_*_→1_does not know its depth *z*_(_*_i-x_*_+1)_*_k_*_→1_, node *s*_(_*_i-x_*_)_*_k_*_→1_ can move to (*x*_(_*_i-x_*_)_*_k_, y*_(_*_i-x_*_)_*_k_, z_sz-end_* + *ε*) and once there establish connection with node *s*_(_*_i-x_*_+1)_*_k_*_→1_ to find out the depth and afterwards compute its optimal location using this information.

Finally, the data sent towards the suface sink travels through the following nodes in the path using hierarchical routing: *s*_(_*_i-x_*_)_*_k_*_→2_, *s*_(_*_i-x_*_)_*_k_*_→1_, *s*_(_*_i-x_*_+1)_*_k_*_→1_,…, *s*_(_*_i-_*_1)_*_k_*_→1_, *s_i_*_→1_, *s_i_*_→2_, *s_i_, s_i_*_+1_,…, and the surface sink.

Generally speaking, the optimal location of the node *s*_(_*_i-x_*_)_*_k_*_→1_ can be determined as follows. We define:

- *u*_(_*_i-x_*_)_*_k_*_→1_ = (*x*_(_*_i-x_*_)_*_k_,y*_(_*_i-x_*_)_*_k_,z*_(_*_i-x_*_)_*_k_*_→1_),∀*k* ∈ *K*, ∀*x* ∈ *N* is the new location of node *s*_(_*_i-x_*_)_*_k_*_→1_.
- *u*_(_*_i-x_*_+1)_*_k_*_→1_ = (*x*_(_*_i-x_*_+1)_*_k_,y*_(_*_i-x_*_+1)_*_k_,z*_(_*_i-x_*_+1)_*_k_*_→1_) is the new already established location of node *s*_(_*_i-x_*_+1)_*_k_*_→1_ for a particular *k* ∈ *K* outside the shadow zone. We consider that node *s*_(_*_i-x_*_+1)_*_k_* was located originally in the shadow zone.
- *u*_(_*_i-x-_*_1)_*_k_* = (*x*_(_*_i-x_*_-1)_*_k_,y*_(_*_i-x-_*_1)_*_k_,z*_(_*_i-x-_*_1)_*_k_*),∀*k* ∈ *K*, ∀*x* ∈ *N* is the location of node *s*_(_*_i-x_*_-1)_*_k_*. We consider that node *s*_(_*_i-x_*_-1)_*_k_* is not located in the shadow zone.

#### P3: Location Optimization Problem

*Given*:*x*_(__*i*__−_*_x_*_)_*_k_, x*_(__*i*__−_*_x_*_+1)_*_k_, x*_(__*i*__−_*_x_*_−1)_*_k_, y*_(__*i*__−_*_x_*_)_*_k_, y*_(__*i*__−_*_x_*_+1)_*_k_, y*_(__*i*__−_*_x_*_−1)_*_k_**z*_(__*i*__−_*_x_*_+1)_*_k_, z*_(__*i*__−_*_x_*_−1)_*_k_, H, R_MAX_,TL_Th_*, α, *χ, A*,*z_sz_*_–_*_start_, z_sz_*_–_*_end_, E_bw_, E_Th_**Find*:*z*_(__*i*__−_*_x_*_)_*_k_*_→1_ ∈ [0, *H*], *r*_(__*i*__−_*_x_*_)_*_k_*_→1 (__*i*__−_*_x_*_+1)_*_k_*_→1_ ∈ *R*^+^*r*_(__*i*__−_*_x_*_−1)_*_k_*_(__*i*__−_*_x_*_)_*_k_*_→1_ ∈ *R*^+^*Objective 1*:*Min*:*TL*_(__*i*__−_*_x_*_)_*_k_*_→1 (__*i*__−_*_x_*_+1)_*_k_*_→1_*Or Objective 2:**Min* max*_k_* (*TL*_(__*i*__−_*_x_*_−1)_*_k_*_(__*i*__−_*_x_*_)_*_k_*_→1_, *TL*_(__*i*__−_*_x_*_)_*_k_*_→1 (__*i*__−_*_x_*_+1)_*_k_*_→1_)*Subject to:*

(16) $$r_{{\left(i-x\right)}_{k}\to 1{\left(i-x+1\right)}_{k}\to 1}=\left\|u_{{\left(i-x+1\right)}_{k}\to 1}{-u}_{{\left(i-x\right)}_{k}\to 1}\right\|,\forall k\in K,\forall x\in N$$

(17) $$r_{{\left(i-x-1\right)}_{k}{\left(i-x\right)}_{k}\to 1}=\left\|u_{{\left(i-x\right)}_{k}\to 1}{-u}_{{\left(i-x-1\right)}_{k}}\right\|,\forall k\in K,\forall x\in N$$

(18) $$r_{{\left(i-x\right)}_{k}\to 1{\left(i-x+1\right)}_{k}\to 1}<R_{\text{MAX}},\forall k\in K,\forall x\in N$$

(19) $$r_{{\left(i-x-1\right)}_{k}{\left(i-x\right)}_{k}\to 1}<R_{\text{MAX}},\forall k\in K,\forall x\in N$$

(20) $$TL_{{\left(i-x\right)}_{k}\to 1{\left(i-x+1\right)}_{k}\to 1}<TL_{Th},\forall k\in K,\forall x\in N$$

(21) $$TL_{{\left(i-x-1\right)}_{k}{\left(i-x\right)}_{k}\to 1}<TL_{Th},\forall k\in K,\forall x\in N$$

(22) $$z_{{\left(i-x\right)}_{k}\to 1}>z_{sz-\text{end}}$$

(23) $$E_{Bw}<E_{Th}$$

We consider two different objectives. The objective function 1 of problem **P3** aims at finding the best location for *s*_(_*_i-x_*_)_*_k_*_→1_ that minimizes the transmission loss of the link *l*_(_*_i-x_*_)_*_k_*_→1(_*_i-x_*_+1)_*_k_*_→1_ and preserves the connectivity with node *s*_(_*_i-x_*_+1)_*_k_*_→1_ preventing from network partition. The objective function 2 of problem **P3** aims at finding the best location for *s*_(_*_i-x_*_)_*_k_*_→1_that minimizes the transmission loss of the link *l*_(_*_i-x_*_)_*_k_*_→1(_*_i-x_*_+1)_*_k_*_→1_ or the link *l*_(_*_i-x_*_-1)_*_k_* _(_*_i-x_*_)_*_k_*_→1_ with the highest transmission loss value and preserves the connectivity with node *s*_(_*_i-x_*_+1)_*_k_*_→1_ and nodes *s*_(_*_i-x-_*_1)_*_k_* preventing from network partition. Objective 1 is valid when the nodes *s*_(_*_i-x-_*_1)_*_k_* are also located in the shadow zone. Otherwise objective 2 should be applied. Constraints (18) and (19) impose that the transmission range should be lower than a threshold to ensure connectivity. Constraints (20) and (21) express that the transmission loss should be lower than a transmission loss threshold. Constraints (19) and (21) are only valid for objective function 2. Constraint (22) imposes that the sensor node *s*_(_*_i-x_*_)_*_k_*_→1_ should be located outside the shadow zone. Constraint (23) states that the energy consumed by the electronically controlled engine to move the sensor to the optimal position should be lower than a threshold.

## 5. Results

Now we study the performance of the proposed scheme under the presence of shadow zones via numerical evaluations. We distinguish between shallow and deep water. The five different channel models considered appear in Section 4. The parameters used in our evaluation are listed in Table 1. They follow the architecture of the commercial off-the-shelf underwater acoustic modem (LinkQuest) UWM3000 for shallow and UWM10000 for deep water [22].

**Figure 6.**
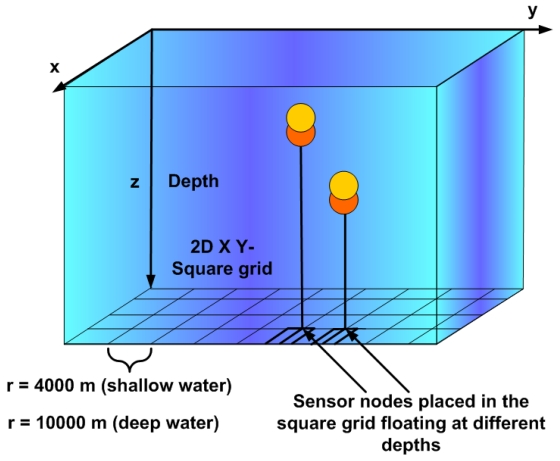
2D Square grid.

The whole seabed is divided into a 2D square grid of equal sizes *r* = 4,000m for shallow water and *r* = 10,000 m for deep water as shown in Figure 6. The sensor nodes are located in a square grid floating at different depths. 25 sensor nodes are deployed in a 3D volume of 16,000 × 16,000 × 100 m^3^ for shallow water and of 40,000 × 40,000 × 5,000 m^3^ for deep water. In shallow water, sensor nodes are located following a hierarchical structure at the depth levels of 25, 50, 75 and 90 m. In deep water, we study the communication between sensor nodes located following a hierarchical structure at the depth levels of 300, 350, 400 and 450 m.

**Figure 7.**
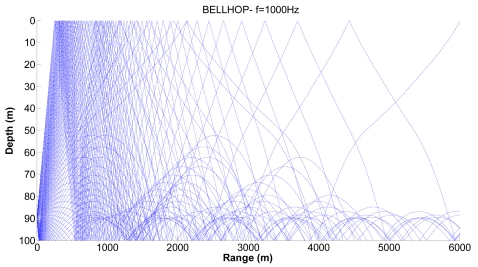
Corresponding ray trace for a source depth of 90 m.

**Figure 8.**
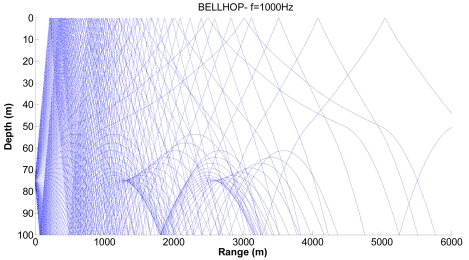
Corresponding ray trace for a source depth of 75 m.

**Figure 9.**
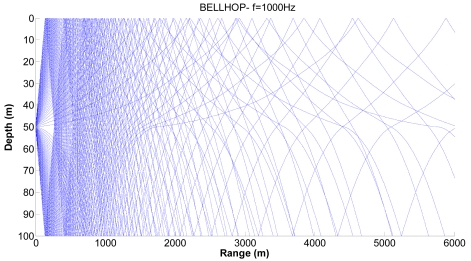
Corresponding ray trace for a source depth of 50 m.

We consider realistic cases where shadow zones can influence the propagation of sensors. Figures 7, 8 and 9 show examples of the propagation paths for the sensor nodes in shallow water. The sound speed profile is based on the measurements conducted at the coast of Kauai, Hawaii [6], with a water depth of 100 m and a frequency of 1 kHz. The rays shown are for launch angles between −20° and 20° with a 1° increment. The corresponding ray traces in Figure 7 for a source depth of 90 m and a receiver depth of 75 m, in Figure 8 for a source depth of 75 m and a receiver depth of 50 m and in Figure 9 for a source depth of 50 m and a receiver depth of 25 m are plotted. Shadow zones appear at ranges around 3 Kms. In our simulations we consider a 2D square grid of equal sizes *r* = 4,000 m because at these ranges it is possible to appreciate the shadow zone effects.

**Figure 10.**
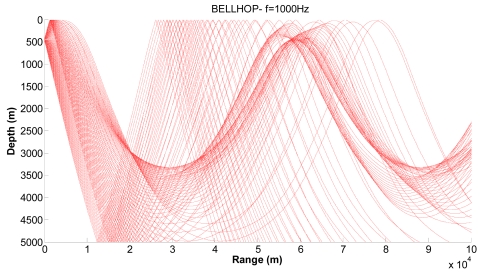
Corresponding ray trace for a source depth of 450 m.

**Figure 11.**
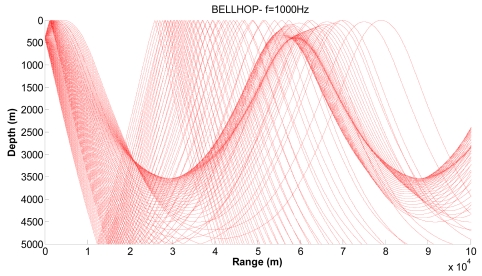
Corresponding ray trace for a source depth of 400 m.

**Figure 12.**
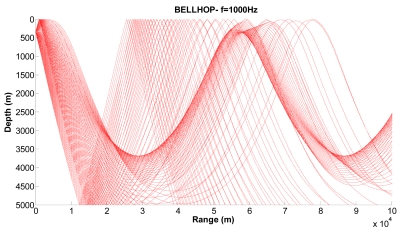
Corresponding ray trace for a source depth of 350 m.

Figures 10, 11 and 12 show examples of the propagation paths for the sensor nodes in deep water. We consider a frequency of 1 kHz and a deep-water (5,000 m) environment with a Munk sound speed profile [2]. The rays shown are for launch angles between −20° and 20° with a 1° increment. The corresponding ray trace in Figure 10 for a source depth of 450 m and a receiver depth of 400 m, in Figure 11 for a source depth of 400 m and a receiver depth of 350 m and in Figure 12 for a source depth of 350 m and a receiver depth of 300 m are plotted. Shadow zones appear at ranges around 10 Kms. In our simulations we consider a 2D square grid of equal sizes *r* = 10,000 m because at these ranges it is possible to appreciate the shadow zone effects.

### Transmisson Loss Threshold

Now we compute the transmission loss threshold *TL_Th_* or maximum propagation loss for properly receiving the transmitted signal given by (9). The link quality in underwater communication is severely affected by multipath phenomena, multipath fading and the refractive properties of the sound channel [3]. As a result, the bit error rates of the acoustic links are often high and efficient error control schemes are needed. The error control schemes automatic repeat request (ARQ), forward error correction (FEC) block codes and FEC convolutional codes have been selected and evaluated in terms of the packet error rate (PER).

According to [3], OFDM transmission with QAM modulation has been used for our analysis. The scalable OFDM with 16-QAM modulation has been successfully tested in the settings with bandwidths 12 kHz, 25 kHz and 50 kHz, leading to data rates about 12 kbps, 25 kbps and 50 kbps respectively [23]. The BER for the modulation scheme 16-QAM is given by:

(24) $$p_{b}^{16\text{QAM}}=\frac{3}{8}\text{erfc}\left(\sqrt{\frac{4}{10}\frac{E_{b}}{N_{0}}}\right),E_{b}/N_{0}=\psi \frac{B_{N}}{R}$$

where *ψ*=10*^ψdB^*^(^*^d^*,*^f^*^)/10^, *B_N_* is the noise bandwidth and *R* is the data rate.

Based on the bit error rate *p_b_*, the PER for the error control schemes can be calculated as follows:

For ARQ, the cyclic redundandy check (CRC) block code detection mechanism is deployed. Assuming detection of all possible packet errors, the PER of a single transmission for a packet of *l* bits is computed as:

(25) $$PER^{\text{CRC}}\left(l\right)=1-{\left(1-p_{b}\right)}^{l}$$

A BCH (Bose, Ray-Chaudhuri, Hocquenghem) and a RS (Reed-Solomon) FEC block code is represented by (*n, k, t*), where *n* is the block length, *k* is the payload length, and *t* is the error correcting capability in bits. For the BCH and RS codes, the block error rate (BLER) is given by:

(26) $$\text{BLER}\left(n,k,t\right)=\sum_{i=t+1}^{n}\left(\begin{array}{c} n\\ i \end{array}\right)p_{b}^{i}{\left(1-p_{b}\right)}^{n-i}$$

Since a packet can be larger than the block length *n*, the PER for BCH and RS block codes is given by:

(27) $$PER^{BC}\left(l,n,k,t\right)=1-{\left(1-\text{BLER}\left(n,k,t\right)\right)}^{\left\lceil \frac{l}{k}\right\rceil }$$

where ⌈*l*/*k*⌉ is the number of blocks required to send *l* bits and ⌈.⌉ is the ceiling function.

For FEC convolutional codes, the PER of a single transmission for a packet of *l* bits is given by:

(28) $$PER^{\text{convolutional}}\left(l,n,k\right)=1-{\left(1-p_{b}\right)}^{\left\lceil \frac{l}{R_{c}}\right\rceil }$$

where *p_b_* is the bit error probability of an encoded data packet of length of length ⌈*l* /*R_c_*⌉ and code rate *R_c_*=*k*/*n*.

**Figure 13.**
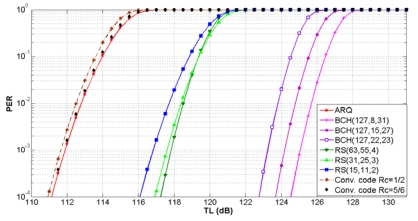
PER as a function of transmission loss for shallow water.

**Figure 14.**
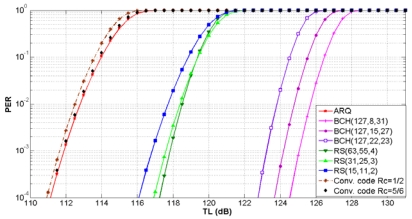
PER as a function of transmission loss for deep water.

The relationship between the PER and *TL_Th_* for ARQ, three BCH, three RS and two convolutional codes is shown in Figure 13 for shallow water and in Figure 14 for deep water. For a target PER, FEC block codes can support *TL_Th_* values higher than ARQ and convolutional codes. We notice that the maximum *TL_Th_* value is increased with the error correcting capability of the FEC block codes and the code rate of the FEC convolutional codes. For a particular *TL_Th_* value, the PER is equal or lower for error control codes in deep water in comparison with shallow water.

The FEC convolutional code with *R_c_* = 1/2 has the lowest *TL_Th_* value. This is the worst case, because the maximum transmission loss is restricted to lower values. Therefore, with this error control scheme for a target PER of 10^−2^ the maximum *TL_Th_* of 112.5 dB for shallow water and 129 dB for deep water have been selected. More details about the performance of error control schemes in UWSNs can be found in [20].

### Energy Consumption in the Sensor Movement

**Figure 15.**
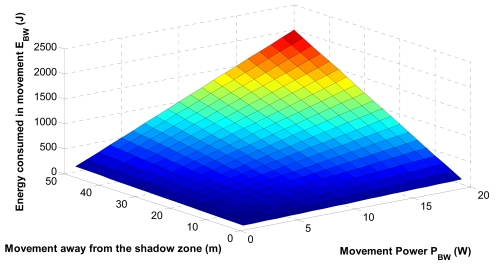
Energy consumption in the sensor displacement.

Now we analyze the energy consumed by the electronically controlled engine to move the sensors affected by the shadow zone to the optimal position. Figure 15 shows the energy consumption in the sensor movement. We observe that the energy consumed is increased with the movement power and the covered distance to reach the optimal location. Since the sensor relocation can be costly in terms of energy consumption, it is limited by the threshold *E_Th_*; this paramenter is estimated analyzing the battery capacity, the required average displacement as well as *P_Bw_*.

### Case 1: Optimal Placement Evaluation

We consider a sensor node *s_i_* located at (*x_i_, y_i_, z_i_*). Node *s_i_*_+1_ is located at (*x_i_* ± *j.r, y_i_* ± *j.r, z_i_*_+1_), *j* ∈ *N, r* = 4,000 m for shallow water, *r* = 10,000 m for deep water, *z_i_*_+1_ < *z_i_*. The acoustic link *l_i i_*_+1_ is affected by a shadow zone with height (*z_sz_*_−_*_end_* − *z_sz_*_−_*_start_*) = 9 m, where *z_i_* > *z_sz_*_−_*_end_* and *z_i_*_+1_ < *z_sz_*_−_*_start_*. Node *s_i_* is uncoupled into sensor nodes *s_i_*_→1_ and *s_i_*_→2_ and the optimal location of node *s_i_*_→2_ has been found using NLP solving the optimization problem **P1**.

**Figure 16.**
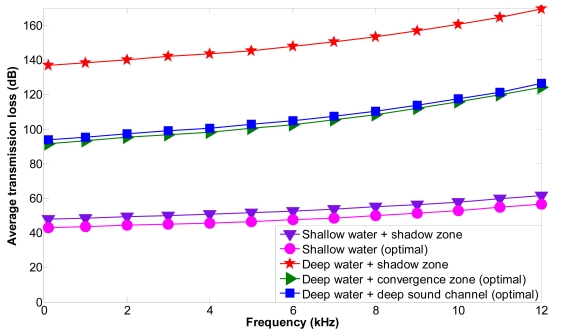
Average transmission loss of a link as a function of frequency in shallow and deep water.

Figure 16 shows the average transmission loss as a function of frequency for shallow and deep water under different propagation phenomena. We have computed the transmission loss for the acoustic link *l_i i_*_+1_ (between *s_i_* and *s_i_*_+1_) affected by the shadow zone (shallow water + shadow zone or deep water + shadow zone). *s_i_* is uncoupled and the optimal location for node *s_i_*_→2_ is found outside the shadow zone using our mathematical model; the transmission loss for the acoustic link *l_i_*_→2 *i*__+1_ (between *s_i_*_→2_ and *s_i_*_+1_) (shallow water (optimal), deep water + convergence zone (optimal) or deep water + deep sound channel (optimal) has also been computed. The average transmission loss is increased when the frequency is increased and is higher for deep water + shadow zone than for shallow water + shadow zone. It is also higher for deep than shallow water. The transmission loss values are higher than the transmission loss threshold for deep water+shadow zone, which means that the transmitted signal will not be properly received. The average transmission loss values are reduced significantly when the optimal locations are computed and the communication between sensor nodes is again restablished outside the shadow zone. The maximum transmission loss improvement is of 4.9 dB for shallow water, of 45.1 dB for deep water + convergence zone and of 42.9 dB for deep water + deep sound channel. Therefore, we can conclude that using our mathematical model deep water + convergence zone shows the best improvement in the diminishment of the transmission loss.

### Case 2: Optimal Placement Evaluation

**Figure 17.**
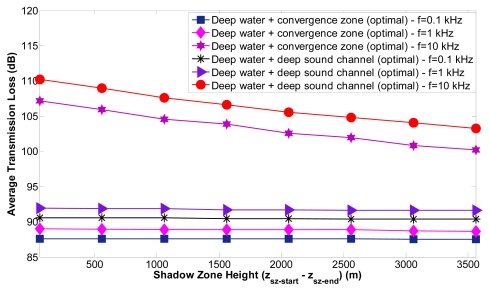
Average transmission loss of a link as a function of the shadow zone height in deep water.

We consider a sensor node *s_i_* located at (*x_i_, y_i_, z_i_*). Node *s_i_*_+1_ is located at (*x_i_* ± *j.r, y_i_* ± *j.r, z_i_*_+1_), *j* ∈ *N, r* = 4,000 m for shallow water, *r* = 10,000 m for deep water, *z_i_*_+1_ < *z_i_*. Nodes *s*_(_*_i-_*_1)_*_k_*, ∀*k* ∈ *K* are located at (*x_i_*±*j.r, y_i_*±*j.r, z*_(__*i*__-1)_*_k_*), *j* ∈ *N, r* = 4,000 m for shallow water, *r* = 10,000 m for deep water, *z*_(__*i*__-1)_*_k_* > *z_i_, k* ∈ *K*. The acoustic links *l_i i_*_+1_ and *l*_(_*_i-_*_1)_*_k i_* are affected by a shadow zone with height (*z_sz_*_−_*_end_* − *z_sz_*_−_*_start_*), where *z_sz_*_−_*_start_* < *z_i_* < *z_sz_*_−_*_end_, z*_(__*i*__-1)_*_k_* > *z_sz_*_−_*_end_, z_i_*_+1_ < *z_sz_*_−_*_start_*. Node *s_i_* is uncoupled into sensor nodes *s_i_*_→1_ and *s_i_*_→2_. The optimal locations of node *s_i_*_→2_ and *s_i_*_→1_ have been found using NLP solving the optimization problems **P1** and **P2**, respectively. Now we evaluate the performance of the proposed scheme for the optimal location of node *s_i_*_→1_ (Case 2). For shallow water the value of *z_sz_*_−_*_end_* varies between 50 and 85 meters, *z_sz_*_−_*_start_* = 40 m and *z*_(__*i*__-1)_*_k_* ∈ (*z_sz_*_−_*_end_*,100]. The average transmission loss of the acoustic link *l*_(_*_i-_*_1)_*_k i_* as a function of the shadow zone height (*z_sz_*_−_*_end_* − *z_sz_*_−_*_start_*) for shallow water and three different frequencies has been computed. The transmission loss of the link *l*_(_*_i-_*_1)_*_k i_* between the node *s*_(_*_i-_*_1)_*_k_*, ∀*k* ∈ *K* and *s_i_*_→1_ with the highest value is minimized for shallow water. The transmission loss values are not affected by the shadow zone height but they are increased with the frequency to 41 dB for *f* =0.1 kHz, 41.6 dB for *f* =1 kHz and 49.3 dB for *f* =10 kHz. Figure 17 shows the average transmission loss of the acoustic link *l*_(_*_i-_*_1)_*_k i_* as a function of the shadow zone height (*z_sz_*_−_*_end_* − *z_sz_*_−_*_start_*) for deep water and three different frequencies. For deep water the value of *z_sz_*_−_*_end_* varies between 500 and 4000 meters, *z_sz_*_−_*_start_* = 440m and *z*_(__*i*__-1)_*_k_* ∈ (*z_sz_*_−_*_end_*,5,000]. The transmission loss of the link *l*_(_*_i-_*_1)_*_k i_* between the node *s*_(_*_i-_*_1)_*_k_*, ∀*k* ∈ *K* and *s_i_*_→1_ with the highest value is minimized for different propagation phenomena (deep water + deep sound channel, deep water + convergence zone). Deep water + convergence zone is the underwater propagation phenomena that suffers lower transmission loss and is more appropriate for underwater communication.

The transmission loss values are especially decreased with the shadow zone height for the frequency of 10 KHz; the reason is that the depth of the optimal location is increased when the shadow zone height is increased (*z_sz_*_−_*_end_* is higher) and the transmission loss decreases when the depth is increased. Otherwise, the transmission loss values for 0.1 KHz and 1 KHz are only very slightly decreased by the shadow zone height and are only significantly increased with the frequency.

### Case 3: Optimal Placement Evaluation

We consider a sensor node *s_i_* located at (*x_i_, y_i_, z_i_*). Nodes *s*_(_*_i-_*_1)_*_k_*, ∀*k* ∈ *K* are located at (*x_i_* ± *j.r, y_i_* ± *j.r, z*_(__*i*__-1)_*_k_*), *j* ∈ *N, r* = 4,000 m for shallow water, *r* = 10,000 m for deep water, *z*_(__*i*__-1)_*_k_* > *z_i_, k* ∈ *K*. Nodes *s*_(_*_i-_*_2)_*_k_*, ∀*k* ∈ *K* are located at (*x_i_* ± *j.r, y_i_* ± *j.r, z*_(__*i*__-2)_*_k_*), *j* ∈ *N, r* = 4,000 m for shallow water, *r* = 10,000 m for deep water, *z*_(__*i*__-2)_*_k_* > *z*_(__*i*__-1)_*_k_, k* ∈ *K*. Nodes *s*_(_*_i-_*_3)_*_k_*, ∀*k* ∈ *K* are located at (*x_i_* ± *j.r, y_i_* ± *j.r, z*_(__*i*__-3)_*_k_*), *j* ∈ *N, r* = 4,000 m for shallow water, *r* = 10,000 m for deep water, *z*_(__*i*__-3)_*_k_* > *z*_(__*i*__-2)_*_k_, k* ∈ *K*. The acoustic links *l*_(_*_i-_*_1)_*_k i_, l*_(_*_i-_*_2)_*_k_* _(_*_i-_*_1)_*_k_, l*_(_*_i-_*_3)_*_k_* _(_*_i-_*_2)_*_k_* are affected by a shadow zone with height (*z_sz_*_−_*_end_* − *z_sz_*_−_*_start_*), where *z_sz_*_−_*_start_* < *z_i_* < *z_sz_*_−_*_end_, z_sz_*_−_*_start_* < *z*_(__*i*__-1)_*_k_* < *z_sz_*_−_*_end_, z_sz_*_−_*_start_* < *z*_(__*i*__-2)_*_k_* < *z_sz_*_−_*_end_, z*_(__*i*__-3)_*_k_* > *z_sz_*_−_*_end_*. Node *s_i_* is uncoupled into sensor nodes *s_i_*_→1_ and *s_i_*_→2_. Nodes *s*_(_*_i-_*_1)_*_k_*, ∀*k* ∈ *K* are uncoupled into sensor nodes *s*_(_*_i-_*_1)_*_k_*_→1_ and *s*_(_*_i-_*_1)_*_k_*_→2_. Nodes *s*_(_*_i-_*_2)_*_k_*, ∀*k* ∈ *K* are uncoupled into sensor nodes *s*_(_*_i-_*_2)_*_k_*_→1_ and *s*_(_*_i-_*_2)_*_k_*_→2_. Node *s_i_*_→1_ is relocated to (*x_i_, y_i_, z_sz-end_* +*ε*), where ε ∈ *R*^+^is a very small value, *z_sz_*_−_*_end_* = 89 m for shallow water and *z_sz_*_−_*_end_* = 4049 m for deep water. The optimal locations of nodes *s*_(_*_i-_*_1)_*_k_*_→1_ have been found with NLP solving the optimization problem **P3** with the objective function 1. The optimal locations of nodes *s*_(_*_i-_*_2)_*_k_*_→1_ have been found with NLP solving the optimization problem **P3** with the objective function 2. In shallow water, sensor nodes are located following a hierarchical structure at the depth levels of 25, 50, 75 and 90 meters. In deep water, sensor nodes are located following a hierarchical structure at the depth levels of 3,900, 3,950, 4,000 and 4,050 meters.

**Figure 18.**
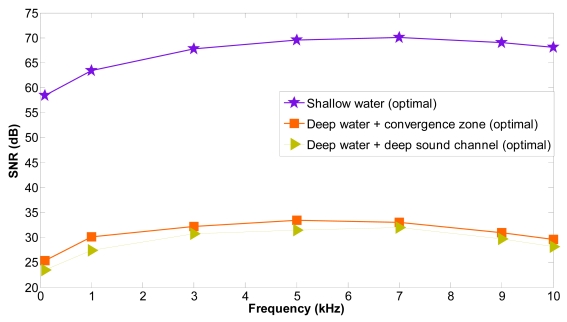
Average SNR as a function of frequency in shallow and deep water.

We consider four hierarchical levels with one node *s_i_* at the lowest level, three nodes *s*_(_*_i-_*_1)_*_k_*, ∀*k* ∈ *K* at the next level, six nodes *s*_(_*_i-_*_2)_*_k_*, ∀*k* ∈ *K* at the next level and 12 nodes *s*_(_*_i-_*_3)_*_k_*, ∀*k* ∈ *K* at the deepest level. Figure 18 shows the average SNR for the acoustic links *l*_(_*_i-_*_1)_*_k i_, l*_(_*_i-_*_2)_*_k_* _(_*_i-_*_1)_*_k_, l*_(_*_i-_*_3)_*_k_* _(_*_i-_*_2)_*_k_* affected by a shadow zone as a function of frequency for shallow and deep water under different propagation phenomena. The average SNR values in the presence of shadow zones are negative, which indicates that the signal is below the noise level and transmission loss, and it can't be properly recovered. They have not been depicted but are decreased when the frequency is increased and are lower for deep water + shadow zone than for shallow water + shadow zone because the transmission loss values are higher for deep water + shadow zone. The average SNR values are increased significantly when the optimal locations are computed and the communication between sensor nodes is again restablished outside the shadow zone. Furthermore, the improved SNR values are maintained for all frequencies. With shallow water the SNR values are better because in shallow water the transmission loss values are lower. The SNR values are higher with deep water + convergence zone than with deep water + deep sound channel. Nevertheless, in all cases the SNR values are positive and they are increased outside the shadow zones. The maximum SNR improvement is very significant: 197.64 dB for shallow water, 352.08 dB for deep water + convergence zone and 350.68 dB for deep water + deep sound channel.

## 6. Conclusions

In this paper, a distributed adaptive topology reorganization scheme that maintains connectivity in multi-hop UWSNs affected by shadow zones has been developed in the context of two Spanish-funded research projects. It alleviates the effect of energy limitations and is able to maintain network connectivity in multi-hop three-dimensional UWSNs in the presence of shadow zones solving a location optimization problem.

Three different cases have been determined according to the number of links in the three-dimensional UWSN affected by the shadow zone. A mathematical model has been developed for each case to find the optimal placement for the sensor nodes, whose ongoing communications are being disturbed.

The average transmission loss values are reduced significantly (especially for deep water + convergence zone) when the optimal locations are computed and the communication between sensor nodes is again reestablished outside the shadow zone. Shallow water shows lower transmission loss values in comparison with the other propagation phenomena. Deep water + convergence zone suffers lower transmission loss values than deep water + deep sound channel and is more appropriate for underwater communication. Only in deep water the transmission loss values are especially decreased with the shadow zone height for the frequency of 10 KHz and only very slightly decreased for the frequencies of 0.1 KHz and 1 KHz; the transmission loss values don't vary with the shadow zone height in shallow water.

The average SNR values in the presence of shadow zones are negative, which indicates that the signal is below the noise level and transmission loss, and it can't be properly recovered. The average SNR values are increased significantly when the optimal locations are computed and the communication between underwater sensors is again reestablished outside the shadow zone. Furthermore, the improved SNR values are maintained for all frequencies. With shallow water the SNR values are better because in shallow water the transmission loss values are lower. The SNR values are higher with deep water + convergence zone than with deep water + deep sound channel. Nevertheless, in all cases the SNR loss values are increased outside the shadow zones.

## Figures and Tables

**Table 1.** Parameter Values.

**Parameter**	**Value**
**Sea depth *H***	Shallow water: 100 m
	Deep water: 5,000 m
**Volume**	Shallow water: 16,000 × 16,000 × 100 m^3^
	Deep water:
	40,000 × 40,000 × 5,000 m^3^
**Number of sensors**	25
**Packet size**	238 bytes
**Data rate**	6 Kbps
***T***	15 °C
***pH***	8
***S***	35 ppt
**Transmission power *P****_t_*	Shallow water: 12 W
	Deep water: 40 W
**Noise level**	47.69 dB
**Speed of sound**	1500 m/s
**Sensor speed**	0.5 m/s
**Movement power *P****_Bw_*	10 W
***Λ***	0.01
***R****_MAX_*	Shallow water: 5,000 m
	Deep water: 11,000 m
***E****_Th_*	1,500 J
***TL****_Th_*	Shallow water: 112.5 dB
	Deep water: 129 dB
